# Complete mitochondrial genome sequence of the phytopathogenic fungi *Sclerotinia sclerotiorum* JX-21

**DOI:** 10.1080/23802359.2016.1219625

**Published:** 2016-09-07

**Authors:** Zhilan Xu, Guoyu Huang, Nannan Song, Jing Wang, Limin Cao, Haiyang Jiang, Ting Ding

**Affiliations:** National Engineering Laboratory of Crop Stress Resistance Breeding, Anhui Agricultural University, Hefei, China

**Keywords:** Ascomycota, Helotiales, *S. sclerotiorum* JX-21, mitochondrial DNA, sequence analysis, phylogenetic analysis

## Abstract

*Sclerotinia sclerotiorum* is one of the most devastating necrotrophic fungal plant pathogens in agriculture causing diseases in over 400 species of plants including important crops and numerous weeds. In this work, the mitochondrial sequence of *S. sclerotiorum* with different strain obtained from the infected stems of *Brassica campestris L.* in Wangjiang County, Anhui Province, China is presented. The mt DNA codes for 14 proteins of the respiratory chain, 1 ribosomal protein, 2 homing endonucleases, 2 rRNAs, 25 tRNAs, and 5 hypothetical proteins ORFs. Phylogenetic analysis with protein-coding gene sequences of reported Ascomycota mt genomes revealed the close relationship of JX-21 with the family of Sclerotiniaceae.

*Sclerotinia sclerotiorum* JX-21 (Fungi, Ascomycota, Ascomycetae, Helotiales, Sclerotiniaceae), is one of the most devastating necrotrophic fungal plant pathogens in agriculture (Bary 1886). It can cause diseases in over 400 species of plants including important crops and numerous weeds (Boland & Hall 1994). The majority of these species are dicotyledonous, such as sunflower, soybean, edible dry bean, and so on, although a number of agriculturally significant monocotyledonous plants are also hosts such as onion and tulip (Bolton et al. 2006). In recent years, 46 Pezizomycotina strains have been sequenced successfully. Most of them are available for members of the Eurotiomycetes and Sordariomycetes with only partial non-annotated genomes or draft annotations available for helotialean species (Duò et al. 2012). Only three mitochondrial genomes belong to members of the Sclerotiniaceae: *S. sclerotiorum*, *S. borealia* (Mardanov et al. 2014), and *Botryotinia fuckeliana*. Here, we resequenced the complete nucleotide sequence of the mt genome of *S. sclerotiorum* with different strains isolate obtained from the infected stems of *Brassica campestris* L., which was collected from March to April in 2012 from Wangjiang County (30°13′ N and 116°68′ E), Anhui Province, China and the strain was stored in the Chinese common microbe bacterial preservation administration centre with the preservation number CGMCC3.18027. The samples were processed within 24 h following the collection and were identified using morphological analysis and ITS sequencing (Guo et al. 2000). The DNA library was sequenced on an IlluminaMiseq in Majorbio and assembled with SOAP denovo v2.04 (Shenzhen, China). The mitochondrial genome of *S. sclerotiorum* strain JX-21 was shown to be a circular DNA molecule of 83,937bp (GenBank KX351425) with an average G + C content of 30.6%. All coding genes are transcribed with different polarities and started with the canonical translation initiation codon-AUG. Fourteen of the 15 typical mitochondrial protein-coding genes are involved in energy and oxidative metabolism (*cox1*, *cox2*, and *cox3*, *cob*, *atp6*, *atp8*, *atp9*, *nad1*, *nad2*, *nad3*, *nad4*, *nad4L*, *nad5*, and *nad6*). The remaining one encoded the 40S ribosomal protein S3 (rps3).

Fourteen intronic ORFs were located in the five protein-coding genes *cox1* (7), *nad5* (3), *nad1* (1), *atp6* (1), *cox3* (1), and *rnl* (1). Ten of the 14 ORFs exhibited similarity in the amino acid sequence to the LAGLIDADG motif and four of them were similar to GIY-YIG motif.

Twenty-five tRNA genes were identified in *S. sclerotiorum* JX-21 mt DNA genome represented 19 amino acids except tRNA^Cys^ and included three copies of tRNA^Met^, tRNA^Ser^, and two copies of tRNA^Ala^, tRNA^Thr^. Single-copy genes encoded the remaining tRNAs. Nineteen tRNAs were grouped into four clusters with 6, 5, 3, and 5 tRNA genes each. Two clusters of 6 and 5 tRNAs were located around *cox1* gene. The remaining two clusters were located around rRNA gene: five around *rnl* and three around *rps3*, while the remaining four tRNA genes occurred, respectively.

The phylogenetic analysis of *S. sclerotiorum* JX-21 was performed by comparison with 15 core mt proteins of other 21 species in Ascomycota (Mardanov et al. 2014) and was constructed by a maximum-likelihood analysis of MEGA 6.0 (Hachioji, Tokyo, Japan) (Tamura et al. 2013) program using 1000 bootstrap replicates. Result shows JX-21 is closely related to *S. sclerotiorum*1980 UF-70, *S. borealia* F-4128, and *B. fuckeliana* B05.10 which are another three members in the family Sclerotiniaceae with high bootstrap value supported (Figure 1). In conclusion, the complete mtDNA of *S. sclerotiorum* JX-21 provides essential and important DNA molecular data for further phylogenetic and evolutionary analysis for Sclerotiniaceae.

**Figure 1.**
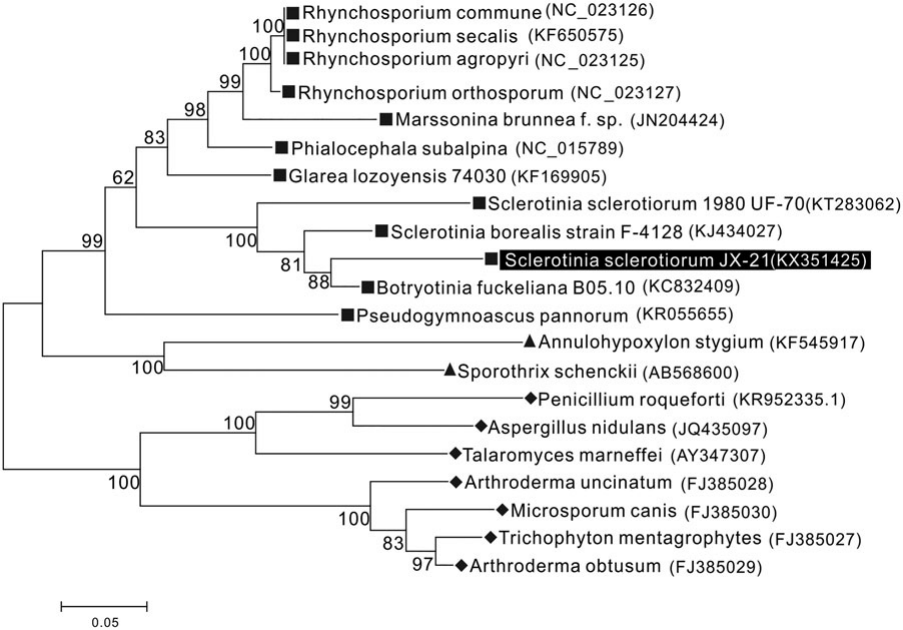
Molecular phylogenetic analysis of *S. sclerotiorum* JX-21 and other Ascomycotina species using the amino acid sequences of 15 common protein-coding genes and the maximum-likelihood method based on the Whelan and Goldman (WAG) model. Numbers at branch nodes are percentages based on 1000 bootstrap resampling. Three classes of filamentous ascomycetes are clearly distinguished as monophyletic groups: square-Leotiomycetes (represented by Helotiales), triangle-Sordariomycetes, and rhombus-Eurotiomycetes. Complete mitogenomes of other species are downloaded from NCBI and the GenBank accession numbers are given in the bracket after the species name.
